# Combination of clinical information and radiomics models for the differentiation of acute simple appendicitis and non simple appendicitis on CT images

**DOI:** 10.1038/s41598-024-52390-z

**Published:** 2024-01-22

**Authors:** Yinming Zhao, Xin Wang, Yaofeng Zhang, Tao Liu, Shuai Zuo, Lie Sun, Junling Zhang, Kexin Wang, Jing Liu

**Affiliations:** 1 Department of Gastrointestinal Surgery, Peking University First Hospital, Beijing, China; 2 Beijing Smart Tree Medical Technology Co. Ltd., Beijing, China; 3 School of Basic Medical Sciences, Capital Medical University Beijing, Beijing, China; 4 Department of Radiology, Peking University First Hospital, Beijing, China

**Keywords:** Computational biology and bioinformatics, Gastroenterology, Medical research

## Abstract

To investigate the radiomics models for the differentiation of simple and non-simple acute appendicitis. This study retrospectively included 334 appendectomy cases (76 simple and 258 non-simple cases) for acute appendicitis. These cases were divided into training (n = 106) and test cohorts (n = 228). A radiomics model was developed using the radiomic features of the appendix area on CT images as the input variables. A CT model was developed using the clinical and CT features as the input variables. A combined model was developed by combining the radiomics model and clinical information. These models were tested, and their performance was evaluated by receiver operating characteristic curves and decision curve analysis (DCA). The variables independently associated with non-simple appendicitis in the combined model were body temperature, age, percentage of neutrophils and Rad-score. The AUC of the combined model was significantly higher than that of the CT model (*P* = 0.041). The AUC of the radiomics model was also higher than that of the CT model but did not reach a level of statistical significance (*P* = 0.053). DCA showed that all three models had a higher net benefit (NB) than the default strategies, and the combined model presented the highest NB. A nomogram of the combined model was developed as the graphical representation of the final model. It is feasible to use the combined information of clinical and CT radiomics models for the differentiation of simple and non-simple acute appendicitis.

## Introduction

Acute appendicitis is a common abdominal disease that can be divided into acute simple appendicitis (SA), acute purulent appendicitis (PA) or suppurative appendicitis, acute gangrenous or perforated appendicitis (GPA), and periappendiceal abscess. Different types may require different treatment methods^1–5^. Recent medical advancements have allowed for safe and effective conservative treatment of acute simple appendicitis with antibiotics^1^. Periappendiceal abscess requiring ultrasound-guided puncture and draining. Acute suppurative or acute gangrenous appendicitis typically requires emergency surgery to prevent perforation and potential life-threatening complications^1–5^. However, it can be challenging for surgeons to determine the pathological type of appendicitis before surgery using imaging modalities such as CT and ultrasound. Previous studies have explored the establishment of predictive models using various clinical information, and there are also studies that investigate the construction of clinical prediction models using CT imaging features or with the combination of CT and clinical information^6,7^. The results show their potential application in distinguishing between uncomplicated and complicated appendicitis^8^. However, there are still certain limitations in their application. Clinical information exhibits considerable variability, and clinical and laboratory test results can change significantly at different stages of the development of appendicitis. The evaluation of CT image features is subjectively performed by physicians, and there is a possibility of inconsistency in the assessment of image features among different radiologists and surgeons. Therefore, the assessment of acute appendicitis still poses some difficulties. Doctors primarily rely on their experience when deciding whether to pursue conservative antibiotic treatment or opt for surgical treatment^2^, leading to a significant level of uncertainty.

Radiomics has been widely used in recent years for image analysis and shows promising results in many fields of diagnosis for gastrointestinal malignancies^9^. It extracts quantitative features from radiological images that cannot be seen by the radiologist’s naked eye. The radiomics data in combination with clinical information can benefit clinical decision processes. Recently, some studies reported that deep learning and radiomics methods could be used to detect acute appendicitis on CT images^10–12^, but few studies have explored the differentiation between the simple and non-simple acute appendicitis^11,13–15^.

The purpose of this study is to explore the application of relatively objective indicators, such as laboratory tests and imaging modality, which could help to make a reasonable treatment plan and reduce unnecessary harm to patients caused by inappropriate treatment.

## Materials and methods

### Data enrollment

This retrospective study was approved by the local Institutional Review Board (IRB) (Peking University First Hospital 2019–169), and informed written consent was waived by the IRB. All methods were performed in accordance with the relevant guidelines and regulations.

All abdominal unenhanced CT images between December 2014 and August 2021 at a local hospital were retrospectively reviewed. The inclusion criteria were (a) appendectomy due to acute appendicitis, (b) available clinical information, and (c) postoperative pathology confirming either simple appendicitis or non-simple appendicitis without periappendiceal abscess. Two surgeons reviewed the surgical and pathological records in consensus. The patients were classified as having non-simple appendicitis when perforated appendicitis or gangrenous appendicitis was present. Patients with diffuse inflammation without perforation, gangrenous, or abscess were classified as having simple appendicitis. The exclusion criteria were as follows: (a) CT images could not be archived from the Picture Archiving and Communication Systems (PACS), (b) CT images did not cover the area of the right lower abdomen, (c) part of the clinical information was missing, (d) CT was examined two weeks before the surgery or acquired after the surgery, and (e) age < 18 years old.

**Figure 1:**
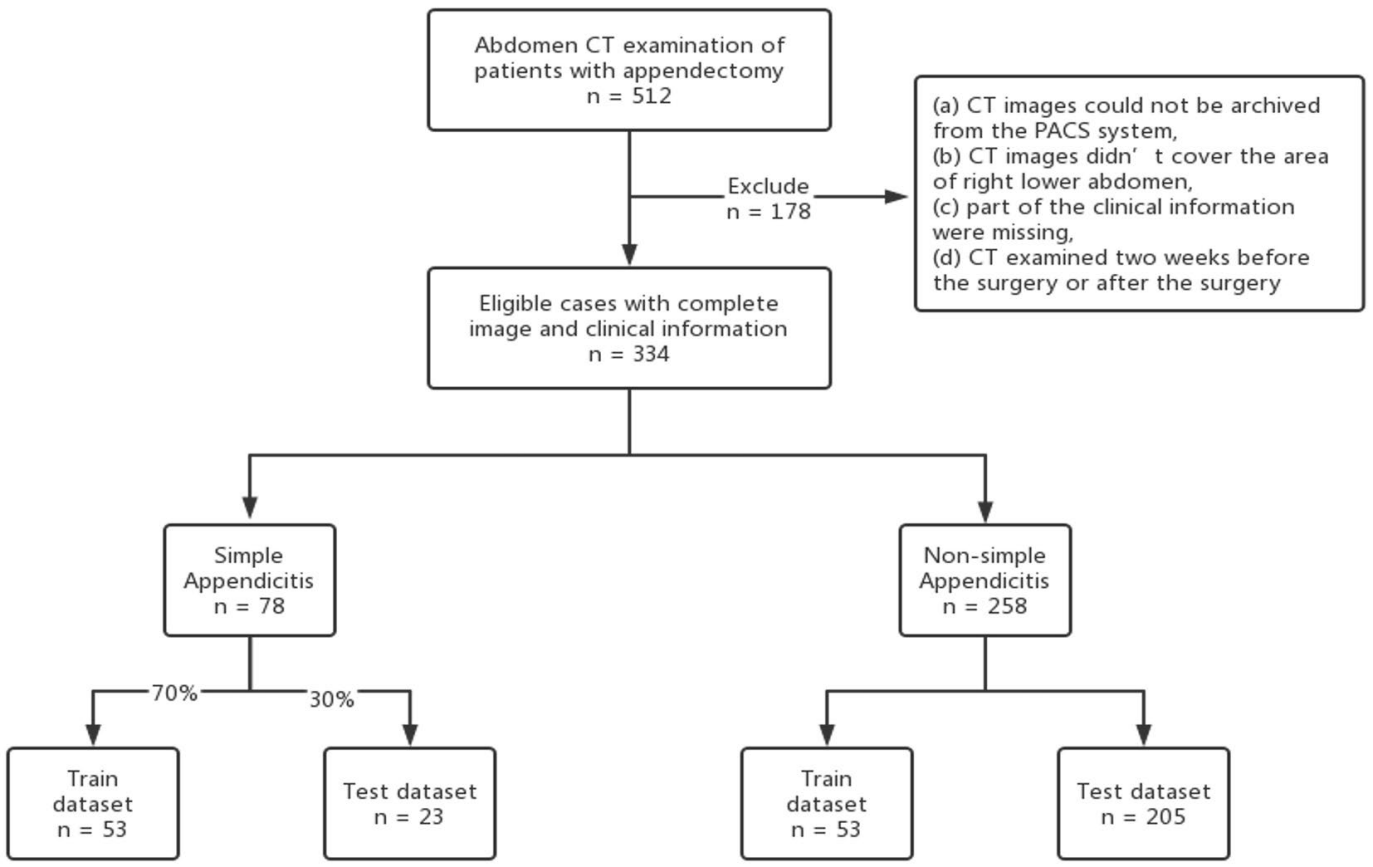
Flow chart of patient enrollment.

The cohort of simple appendicitis patients was randomly assigned to the training dataset (n = 53) and test dataset (n = 23) at a ratio of 7:3. For the non-simple appendicitis cohort, 53 cases were randomly assigned to the training dataset, and the other 205 cases were assigned to the test dataset (Fig. 1).

### Information collection from the cohort

#### Clinical information

The body temperature on admission of patients was recorded. The lab test results of C-reactive protein (CRP), bilirubin, white blood cell (WBC), and percentage of neutrophils (NE%) were also recorded from the electronic medical record system. The laboratory tests were obtained on the same day as the CT scan.

#### CT acquisition parameters

Abdominal CT images were acquired from seven CT scanners. The detailed scanning parameters are shown in Table 1.

**Table 1:** Image acquisition protocols for abdominal CT.

	Test	Train	Overall
	(N = 228)	(N = 106)	(N = 334)
Manufacture			
GE MEDICAL SYSTEMS	221 (96.9%)	103 (97.2%)	324 (97.0%)
NMS	1 (0.4%)	0 (0%)	1 (0.3%)
Philips	3 (1.3%)	2 (1.9%)	5 (1.5%)
SIEMENS	3 (1.3%)	1 (0.9%)	4 (1.2%)
Model Name			
Brilliance 64	1 (0.4%)	0 (0%)	1 (0.3%)
Discovery CT750 HD	10 (4.4%)	0 (0%)	10 (3.0%)
iCT 256	2 (0.9%)	2 (1.9%)	4 (1.2%)
LightSpeed VCT	208 (91.2%)	103 (97.2%)	311 (93.1%)
NeuViz Glory	1 (0.4%)	0 (0%)	1 (0.3%)
Optima CT620	3 (1.3%)	0 (0%)	3 (0.9%)
SOMATOM Definition Flash	3 (1.3%)	1 (0.9%)	4 (1.2%)
Reconstruction Diameter			
Median [Min, Max]	391 [330, 500]	392 [315, 500]	391 [315, 500]
Slice Thickness			
Median [Min, Max]	1.25 [1.00, 2.00]	1.25 [1.00, 1.25]	1.25 [1.00, 2.00]
Slice Spacing			
Median [Min, Max]	1.25 [− 1.00, 5.00]	1.25 [1.00, 1.25]	1.25 [− 1.00, 5.00]
Pixel Spacing			
Median [Min, Max]	0.764 [0.645, 0.977]	0.765 [0.615, 0.977]	0.764 [0.615, 0.977]

#### Assessment of the CT image

The CT images were reviewed and checked by two experienced radiologists (15 and 29 years of experience in abdominal radiology). The following findings were recorded: perforation, abscess, peritonitis, appendix wall thickening, cecum wall thickening, fecalith, appendiceal intramural and extraluminal air, surrounding strand, pneumoperitoneum, and ileocecal lymph node enlargement. The width of the appendix was also measured and recorded. For measurement of the width of the appendix, we used the curved reconstruction method on the CT post-processing workstation to locate the centerline of the appendix and reconstruct the full-length image of the appendix. Then, we identified the location where the appendix was most thickened and measured its short axis to determine the width of the appendix.

#### Region of interest (ROI)

**Figure 2:**
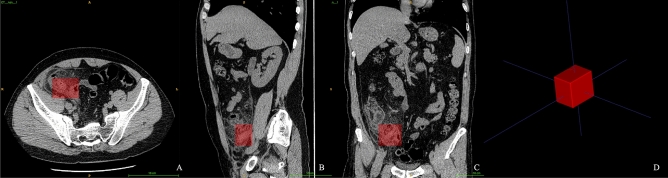
Region of interest of the models. A rectangle-shaped ROI was annotated, covering the areas of the end of the cecum and appendix. The ROI is shown in axial (**A**), sagittal (**B**), and coronal (**C**) planes and viewed three dimensionally (**D**).

The appendix area on the CT images was manually labeled by two readers (reader A, radiologist with 11 years of experience, reader B, intern in radiology training) and validated by an experienced radiologist (with 29 years of experience) with ITK-Snap software (http://www.itksnap.org). A 3D cube shape of the ROI was annotated at the end of the cecum and the appendix area^12^ (Fig. 2).

For measurement of the ROI of the appendix area, we examined the appendix area layer by layer on axial, coronal, and sagittal reconstructed images to identify key points marking the upper, lower, left, right, inner, and outer boundaries of the appendix. The ROI should include the root of the cecum and its inner lower part. A bounding box was generated using NumPy based on these key points, which served as the ROI for the radiomics study in this research. The length, width, and height of the rectangular ROI, volume, and average CT value of all voxels within the ROI were obtained using NumPy and SimpleITK. These measured values were subsequently used for statistical analysis.

### Development of the Models

**Figure 3:**
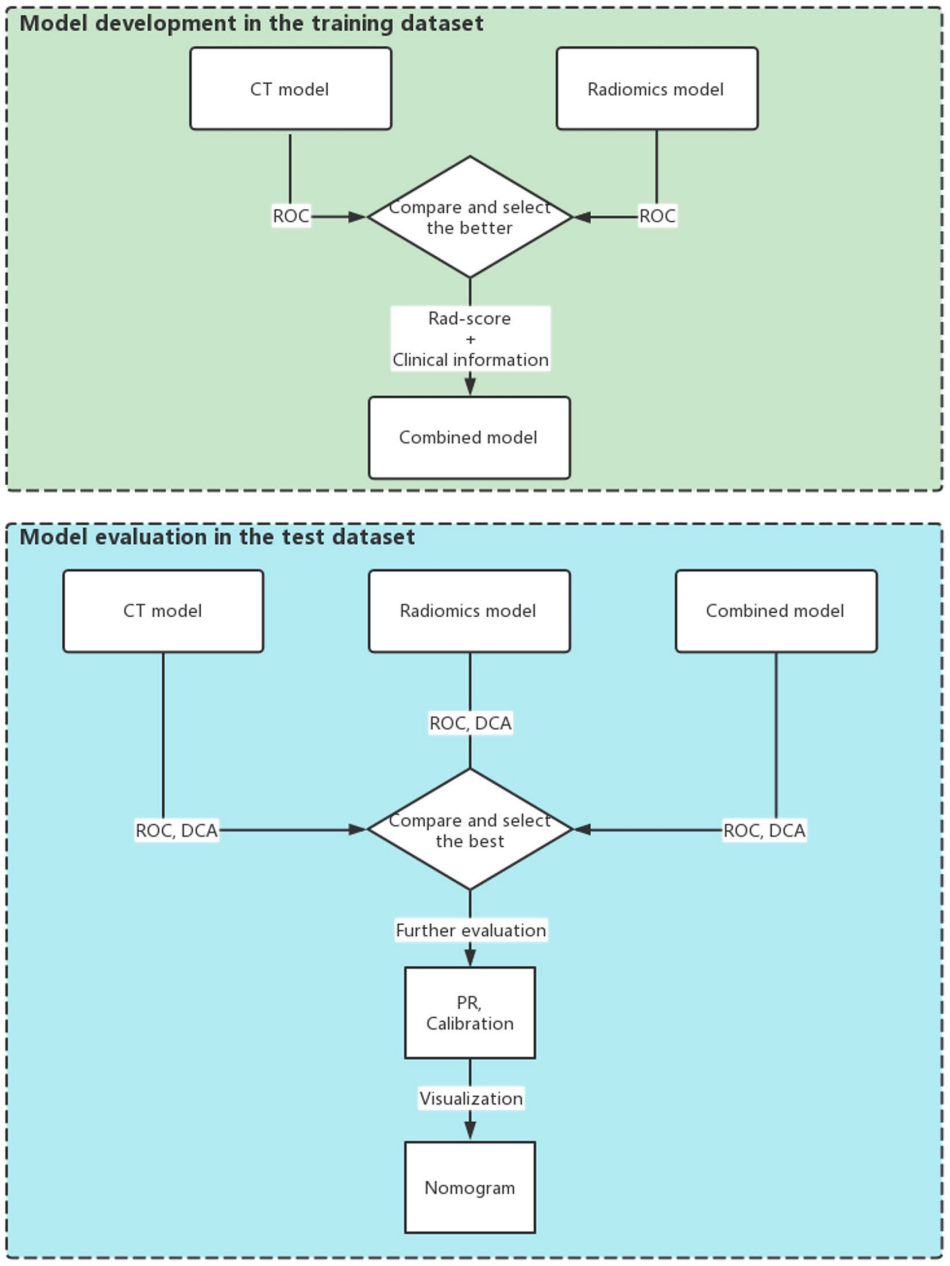
Study workflow. Three models were developed in the training dataset. First, the CT model and the radiomics model were developed. The better one was chosen, and its output, named the “Rad-score”, was taken to join the clinical information for the development of the combined model. Then, the three models were evaluated in the test dataset. ROC and DCA analyses were performed, and the best model was chosen for further evaluation with precision-recall curve and calibration curve analyses. Finally, a nomogram was developed as the graphical representation of our best model.

Three models were developed in the training cohort, including a CT model, a radiomics model and a combined model (Fig. 3). R language (version 4.1.3) was used for model development.

First, we developed the CT model and the radiomics model. For the CT model, the dependent variables were the qualitative and quantitative features that were defined by the radiologists’ visual assessment of the CT images. For the radiomics model, the dependent variables were the radiomic features that were derived by the PyRadiomics package.

Second, after the comparison of the CT model and the radiomics model, the better model was chosen to combine the clinical information and to develop the combined model.

#### Development of the CT MODEL

A multivariable logistic regression model was built using the training cohort. The model started with all covariates of CT features listed in the abovementioned visual assessment of the CT images. Univariable analysis was performed to observe the independent impact of each predictor variable on the non-simple appendicitis status. Then, multivariable analysis was performed using a bi-directional stepwise algorithm to choose the predictor variables for the final multivariable model by the Akaike information criterion (AIC). The “glm” R package was used to conduct logistic regression analysis and stepwise predictor selection from the “stats” package and the “autoReg” package.

#### Development of the radiomics model

The ROIs were preprocessed to a uniform size. The image features were extracted by the PyRadiomics package of Python (https://pyradiomics.readthedocs.io/en/latest/features.html). Fourteen shape-based features, 18 first-order features, and 70 texture features were calculated. The Z score normalization method was applied to rescale the features. Pearson correlation coefficients (PCCs) were calculated, and the features with PCC > 0.99 were dropped to avoid multicollinearity. LASSO was used to select the features to fit the radiomics model. Cross-validation was used to select the best model. During the fivefold cross-validation, we identified the minimum lambda value, which was 0.065. It was then employed to train the final radiomics model. This approach ensures the selection of an optimal level of regularization, enhancing the model's generalization performance. The predicted probability (Rad-score) by the LASSO logistic regression classifier was used to evaluate the efficacy of the radiomics model and was also used as the input of the combined model. The “glmnet” R package was used to train the LASSO model.

To study the reproducibility of the radiomic features, 40 patients (20 with simple appendicitis and 20 with non-simple appendicitis) in the training cohort were randomly selected and labeled again by reader A and reader B. Intraclass correlation coefficients (ICCs) were calculated from a two-way random effects model to determine the inter- and intraobserver reliability. Only radiomics features that had excellent reliability (ICC > 0.85) were considered robust.

#### Development of the combined model

A multivariable logistic regression model was built in combination with the clinical information and the Rad-score. The model fitting process is the same as the development of the CT model.

### Evaluation of the models

During cross-validation, the AUC was calculated for both CT and radiomic models, and the best model was selected. Subsequently, the AUCs of the combined model were tested using the same folds. The models were then compared based on their AUCs in both the training cohort and the test cohort. The predictive accuracy of the models was assessed using ROC. For the decision curve analysis (DCA) was calculated in the test cohort. Bootstrapping using 1,000 repetitions was used to calculate the standardized net benefit by the probability threshold. Finally, a nomogram was developed as the graphical representation of our best model.

### Statistical analysis

Statistical analyses were performed using R4.1.3 software. Continuous variables conforming to a normal distribution are expressed as the mean ± standard deviation, and those not conforming to a normal distribution are expressed as the median [Q1, Q3]. Categorical variables are expressed as frequencies and percentages. The Kolmogorov‒Smirnov test was used to test for a normal distribution. The chi-square and Fisher’s exact tests were used to assess associations between categorical variables. The Mann‒Whitney U test was used to compare the differences between two groups when the sample distribution was not normally distributed. The Kruskal‒Wallis test was used to compare the differences between multiple groups in situations when the assumptions of parametric tests were not met. The DeLong test was performed on AUCs among different models. P value less than 0.05 was considered a statistically significant difference.

### Ethical approval

Ethical approval was obtained from the institutional review board (Peking University First Hospital Ethics Committee) (2019–169), and informed written consent was waived by the IRB. All methods were performed in accordance with the relevant guidelines and regulations.

## Results

### Clinical and CT features of appendicitis

A total of 334 eligible cases were included in this study, consisting of 76 cases of simple appendicitis and 258 cases of non-simple appendicitis. The median age for simple appendicitis was 39.0 years [Q1 = 29.0, Q3 = 55.0], while for non-simple appendicitis it was 44.0 years [Q1 = 34.0, Q3 = 60.8]. The overall median age was 43.0 years [Q1 = 32.0, Q3 = 58.8]. The gender distribution for simple appendicitis included 38 females (50.0%) and 38 males (50.0%). In non-simple appendicitis, 109 females (42.2%) and 149 males (57.8%) were observed. In the entire cohort, 147 females (44.0%) and 187 males (56.0%) were present.

The median time interval between the laboratory tests, CT scan and surgery for the 334 patients was 0 [Q1 = 0, Q3 = 1]. Among the 334 patients, 319 had a time interval of ≤ 24 h, 14 patients had an interval of 2–3 days, 1 patient had an interval of 6 days, 3 patients had an interval of 7 days, and 1 patient had an interval of 14 days.

The clinical and CT features of the two types of appendicitis are shown in Tables 2 and 3.

**Table 2:** Clinical characteristics of the patients with simple and non-simple appendicitis.

	Simple Appendicitis	non-Simple Appendicitis	Overall	*P* value
	(N = 76)	(N = 258)	(N = 334)	
Age (years)				
Median [Q1,Q3]	39.0 [29.0,55.0]	44.0 [34.0,60.8]	43.0 [32.0,58.8]	0.104
Gender				
Female	38 (50.0%)	109 (42.2%)	147 (44.0%)	0.489
Male	38 (50.0%)	149 (57.8%)	187 (56.0%)	
Body Temperature (℃)				
Median [Q1,Q3]	36.6 [36.4,37.0]	37.4 [37.0,37.8]	37.2 [36.8,37.8]	< 0.001
CRP (mg/L)				
Median [Q1,Q3]	3.00 [2.00,8.25]	36.0 [5.00,72.8]	15.0 [4.00,67.0]	< 0.001
WBC (× 10^9^/L)				
Median [Q1,Q3]	9.80 [6.88,13.6]	13.3 [11.3,15.5]	13.0 [10.1,15.2]	< 0.001
NE (%)				
Median [Q1,Q3]	77.5 [66.8,88.2]	85.7 [78.0,90.0]	85.0 [76.0,90.0]	< 0.001
Bilirubin (umol/L)				
Median [Q1,Q3]	15.8 [12.8,21.5]	19.2 [15.2,27.2]	18.9 [14.3,26.0]	0.002

**Table 3:** CT features in simple and non-simple appendicitis.

	Simple Appendicitis	non-Simple Appendicitis	Overall	*P* value
	(N = 76)	(N = 258)	(N = 334)	
Perforation				
Absent	74 (97.4%)	238 (92.2%)	312 (93.4%)	0.286
Present	2 (2.6%)	20 (7.8%)	22 (6.6%)	
Abscess				
Absent	74 (97.4%)	254 (98.4%)	328 (98.2%)	0.823
Present	2 (2.6%)	4 (1.6%)	6 (1.8%)	
Peritonitis				
Absent	67 (88.2%)	201 (77.9%)	268 (80.2%)	0.143
Present	9 (11.8%)	57 (22.1%)	66 (19.8%)	
Appendix Wall Thickening				
Absent	31 (40.8%)	80 (31.0%)	111 (33.2%)	0.282
Present	45 (59.2%)	178 (69.0%)	223 (66.8%)	
Fecal Retention				
Absent	53 (69.7%)	140 (54.3%)	193 (57.8%)	0.0561
Present	23 (30.3%)	118 (45.7%)	141 (42.2%)	
Thickening of Cecum				
Absent	56 (73.7%)	92 (35.7%)	148 (44.3%)	< 0.001
Present	20 (26.3%)	166 (64.3%)	186 (55.7%)	
Thickness of Appendix (mm)				
Median [QPresent,Q3]	1.10 [0.900,1.30]	1.20 [1.10,1.40]	1.20 [1.10,1.40]	< 0.001
Surrounding Strand				
Absent	22 (28.9%)	14 (5.4%)	36 (10.8%)	< 0.001
Present	54 (71.1%)	244 (94.6%)	298 (89.2%)	
Appendiceal Intraluminal Gas				
Absent	76 (100%)	227 (88.0%)	303 (90.7%)	0.007
Present	0 (0%)	31 (12.0%)	31 (9.3%)	
Pneumoperitoneum				
Absent	76 (100%)	232 (89.9%)	308 (92.2%)	0.016
Present	0 (0%)	26 (10.1%)	26 (7.8%)	
Ileocecal Lymph Node				
Absent	36 (47.4%)	77 (29.8%)	113 (33.8%)	0.018
Present	40 (52.6%)	181 (70.2%)	221 (66.2%)	
Overall Impression				
No appendicitis according to CT	5 (6.6%)	2 (0.8%)	7 (2.1%)	0.036
Simple appendicitis	67 (88.2%)	234 (90.7%)	301 (90.1%)	
Non-simple appendicitis	4 (5.3%)	22 (8.5%)	26 (7.8%)	

The characteristics were assessed for the entire cohort and compared using Mann‒Whitney U tests and chi-square tests for continuous and categorical variables, respectively. No significant difference was found between simple and non-simple appendicitis in terms of age, sex, visual assessment of perforation, abscess, peritonitis, or appendix wall thickening (*P* > 0.05). Statistically significant differences existed between the simple and non-simple appendicitis groups in body temperature, CRP, WBC, NE%, bilirubin, thickness of the appendix, visual assessment of fecalith, thickening of the cecum, surrounding strand, appendiceal intramural air and extraluminal air, pneumoperitoneum, ileocecal lymph node enlargement, and the overall impression of the CT images (*P* < 0.05).

### Parameters of the model

#### Results of the CT model

**Figure 4:**
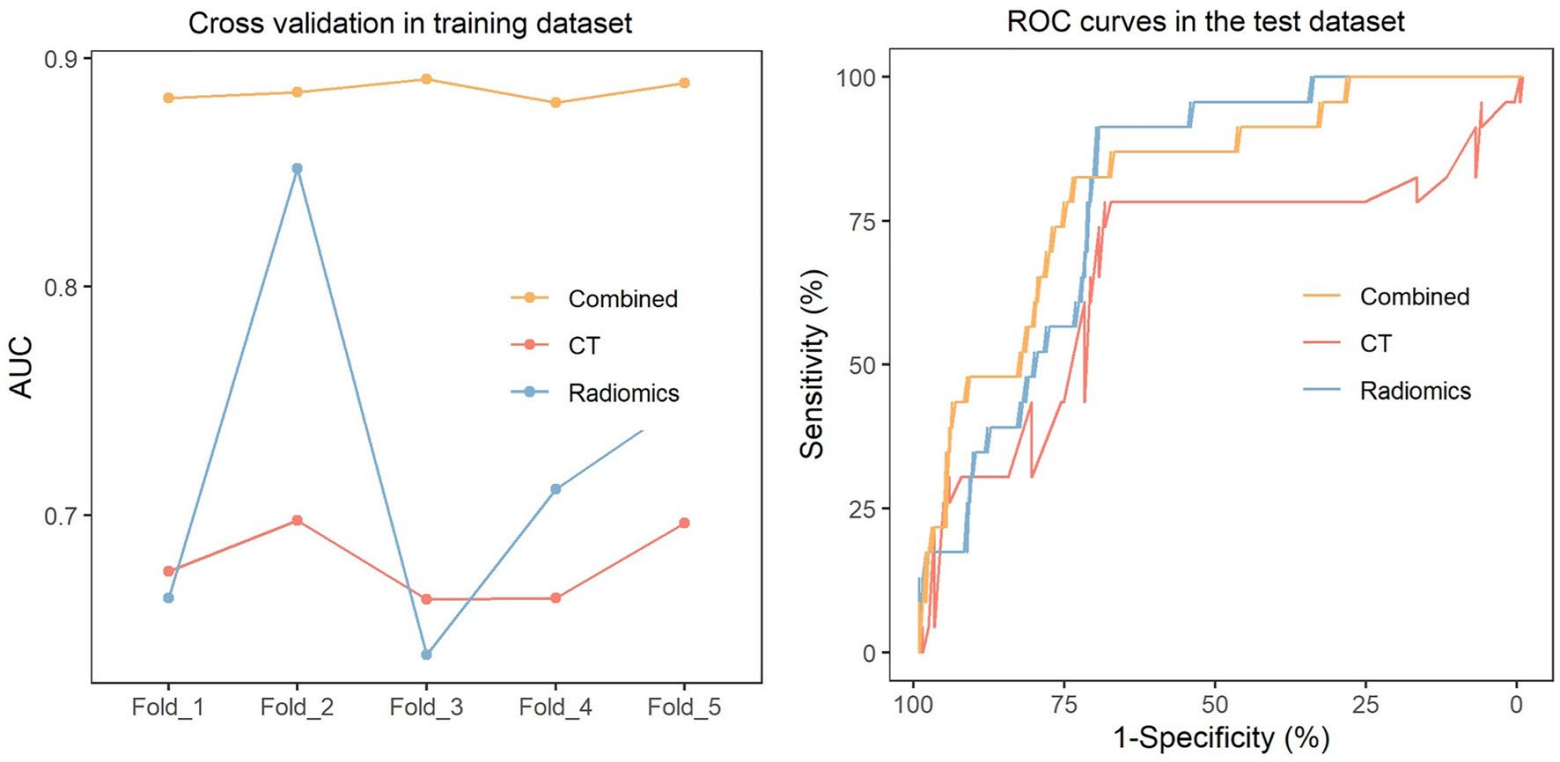
Cross-validation in the training dataset and ROC curves in the test dataset. (**A**) The process involved fivefold cross-validation in the training set for the three models, using the same data subdivision. The AUCs of the CT model were as follows: 0.675, 0.698, 0.663, 0.664, and 0.697, with an average AUC of 0.679. The AUCs of the radiomic model were 0.664, 0.852, 0.639, 0.712, and 0.750, with an average AUC of 0.723. The AUCs of the combined model were 0.883, 0.885, 0.891, 0.881, and 0.889, with an average AUC of 0.886. (**B**) ROC curves of the CT model, radiomics model, and combined model in the test dataset. There was no significant difference in the AUCs between the combined model and the radiomics model according to the DeLong test (0.817 vs. 0.804, *P* = 0.808). The AUC of the combined model was significantly higher than that of the CT model (0.817 vs. 0.669, *P* = 0.041). The AUC of the radiomics model was also higher than that of the CT model but did not reach a level of statistical significance (0.804 vs. 0.669, *P* = 0.053).

Univariable and multivariable logistic regression were performed, and the odds ratios of the variables are shown in the Supplementary material. The CT image findings independently associated with non-simple appendicitis and included in the final model were strand near the appendix and thickening of the cecum wall. The AUC of the CT regression model was 0.604 (95% CI 0.494 ~ 0.713, Fig. 4A).

#### Results of the Radiomics Model

The LASSO regression function included five arguments, i.e., three shape-based features and two texture features (Supplementary material). After obtaining the radiomics model, we input the patient’s ROI data into the model, and the model outputs a predicted value, which is a numeric value between 0 and 1. This predicted value represents the radiomics model's prediction rate for whether the patient has non-simple appendicitis, which is named the Rad-score. The higher the Rad-score is, the greater the likelihood that the patient has non-simple appendicitis. The predictive ability of the Rad-score was evaluated using the AUC with pathological results as the reference standard. Additionally, the Rad-score was fitted together with other indicators as input parameters to form a combined model. The AUC of the Rad-score of the radiomics model was 0.766 (95% CI 0.674 ~ 0.858, Fig. 4A).

#### Results of the combined model

The DeLong test was performed to compare the AUCs of the radiomics model and the CT model. The AUC of the radiomics model was higher than that of the CT model, with statistical significance (*P* = 0.006). The cross-validated AUC of the radiomics model was higher than that of the CT model; hence, the predictive probability of radiomics model (Rad-score) was incorporated into the combined model. Univariable and multivariable logistic regression was performed to assess variables associated with non-simple appendicitis, and a summary of the results is displayed in Table 4. The variables independently associated with non-simple appendicitis and included in the final model were body temperature, age, neutrophil percentage, and Rad-score.

**Table 4:** Odds ratios in univariate and multivariate logistic regression analyses of the combined model.

Variables	Univariable regression		Multivariable regression	
	OR (95% CI)	*P* value	OR (95% CI)	*P* value
Gender	1.461 (0.681, 3.163)	0.332		
Bilirubin (umol/L)	1.045 (1.000, 1.098)	0.062		
CRP (mg/L)	1.014 (1.003, 1.027)	0.023		
WBC (× 10^9^/L)	1.182 (1.074, 1.311)	< 0.001		
Body Temperature (℃)	8.993 (3.964, 23.438)	< 0.001	7.377 (2.822, 22.517)	0.000
Age (years)	1.037 (1.011, 1.066)	0.006	1.051 (1.016, 1.093)	0.007
NE (%)	1.072 (1.032, 1.12)	< 0.001	1.046 (0.996, 1.103)	0.079
Rad-score	2.627 × 10^6^ (1.175 × 10^3^, 2.386 × 10^10^)	< 0.001	1.545 × 10^5^ (13.903, 5.257 × 10^9^)	0.017

### Evaluation of the models

**Figure 5:**
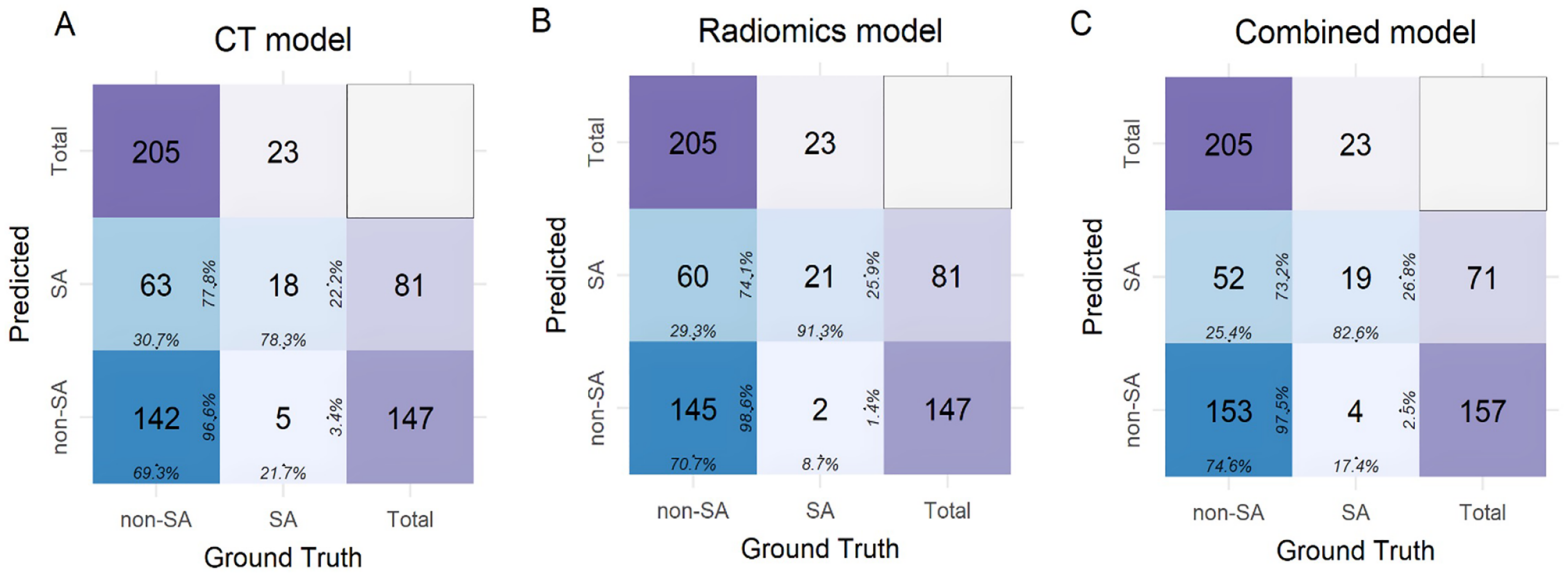
Confusion matrices of the models. SA: simple appendicitis, non-SA: nonsimple appendicitis. Confusion matrix displays the performance of the CT model (**A**), radiomics model (**B**), and the combined model (**C**). Among the 205 non-SA cases, the combined model detected 153 true positive cases, surpassing the CT model (142) and the radiomics model (145).

The AUCs and other evaluation metrics of the models in both the training and test cohorts are shown in Table 5. The comparison of the AUCs with the DeLong test is presented in Table 6. The confusion matrices of the three models in the test set are shown in Fig. 5.

**Table 5:** Classification metrics of the three models in the training and test datasets.

	CT_train	CT_test	Radiomics_train	Radiomics_test	Combined_train	Combined_test
AUC (95% CI)	0.604 (0.494, 0.713)	0.669 (0.528, 0.809)	0.766 (0.674, 0.858)	0.804 (0.730, 0.878)	0.884 (0.820, 0.949)	0.817 (0.728, 0.905)
Cutoff	0.569	0.515	0.504	0.483	0.446	0.452
Accuracy (95% CI)	0.642 (0.637, 0.646)	0.702 (0.700, 0.704)	0.726 (0.723, 0.730)	0.728 (0.726, 0.730)	0.858 (0.856, 0.861)	0.754 (0.753, 0.756)
Sensitivity (95% CI)	0.566 (0.433, 0.699)	0.693 (0.630, 0.756)	0.660 (0.533, 0.788)	0.707 (0.645, 0.770)	0.887 (0.801, 0.972)	0.746 (0.687, 0.806)
Specificity (95% CI)	0.717 (0.596, 0.838)	0.783 (0.614, 0.951)	0.792 (0.683, 0.902)	0.913 (0.798, 1.000)	0.830 (0.729, 0.931)	0.826 (0.671, 0.981)
Positive predict value (95% CI)	0.667 (0.529, 0.804)	0.966 (0.937, 0.995)	0.761 (0.638, 0.884)	0.986 (0.968, 1.005)	0.839 (0.743, 0.935)	0.975 (0.950, 0.999)
Negative predict value (95% CI)	0.623 (0.501, 0.745)	0.222 (0.132, 0.313)	0.700(0.584, 0.816)	0.259 (0.164, 0.355)	0.880 (0.790, 0.970)	0.268 (0.165, 0.371)
Positive likelihood ratio (95% CI)	2.000 (1.226, 3.262)	3.186 (1.460, 6.956)	3.182 (1.817, 5.572)	8.134 (2.157, 30.670)	5.222 (2.857, 9.544)	4.291 (1.755, 10.495)
Negative likelihood ratio (95% CI)	0.605 (0.426, 0.86)	0.393 (0.292, 0.529)	0.429 (0.287, 0.639)	0.321 (0.250, 0.411)	0.136 (0.064, 0.293)	0.307 (0.227, 0.415)

*CT_train: CT model evaluated in the training cohort. CT_test: CT model evaluated in the test cohort. Radiomics_train: radiomics model evaluated in the training cohort. Radiomics_test: radiomics model evaluated in the test cohort. Combined_train: the combined model evaluated in the training cohort. Combined_test: combined model evaluated in the test cohort.

**Table 6:** DeLong test of the three models in the training and test datasets.

	CT_train	CT_test	Radiomics_train	Radiomics_test	Combined_train	Combined_test
CT_train	NA	0.476	0.006	0.003	< 0.001	0.003
CT_test	0.476	NA	0.256	0.053	0.007	0.041
Radiomics_train	0.006	0.256	NA	0.531	0.003	0.437
Radiomics_test	0.003	0.053	0.531	NA	0.110	0.808
Combined_train	< 0.001	0.007	0.003	0.110	NA	0.228
Combined_test	0.003	0.041	0.437	0.808	0.228	NA

*CT_train: CT model evaluated in the training cohort. CT_test: CT model evaluated in the test cohort. Radiomics_train: radiomics model evaluated in the training cohort. Radiomics_test: radiomics model evaluated in the test cohort. Combined_train: the combined model evaluated in the training cohort. Combined_test: combined model evaluated in the test cohort.

No statistically significant differences were observed in the AUCs of the same model between the training and testing sets (all *P *> 0.05), indicating that the models have some degree of generalization capability. When comparing the combined model with other models, the Combined_train AUC was significantly higher than that of CT_train, CT_test, and Radiomics_train (all *P *< 0.05), but the difference from Radiomics_test was not statistically significant (*P *= 0.110). The combined_test AUC was significantly higher than that of CT_train and CT_test (both *P *< 0.05), but there were no statistically significant differences between Radiomics_train and Radiomics_test (both *P *> 0.05).

ROC analysis showed (Fig. 4B) that there was no significant difference in the AUCs between the combined model and the radiomics model according to the DeLong test (0.817 vs. 0.804, *P *= 0.808). The AUC of the combined model was significantly higher than that of the CT model (0.817 vs. 0.669, *P* = 0.041). The AUC of the radiomics model was also higher than that of the CT model but did not reach a level of statistical significance (0.804 vs. 0.669, *P* = 0.053). The PR curve and the calibration curve are shown in the Supplementary material.

**Figure 6:**
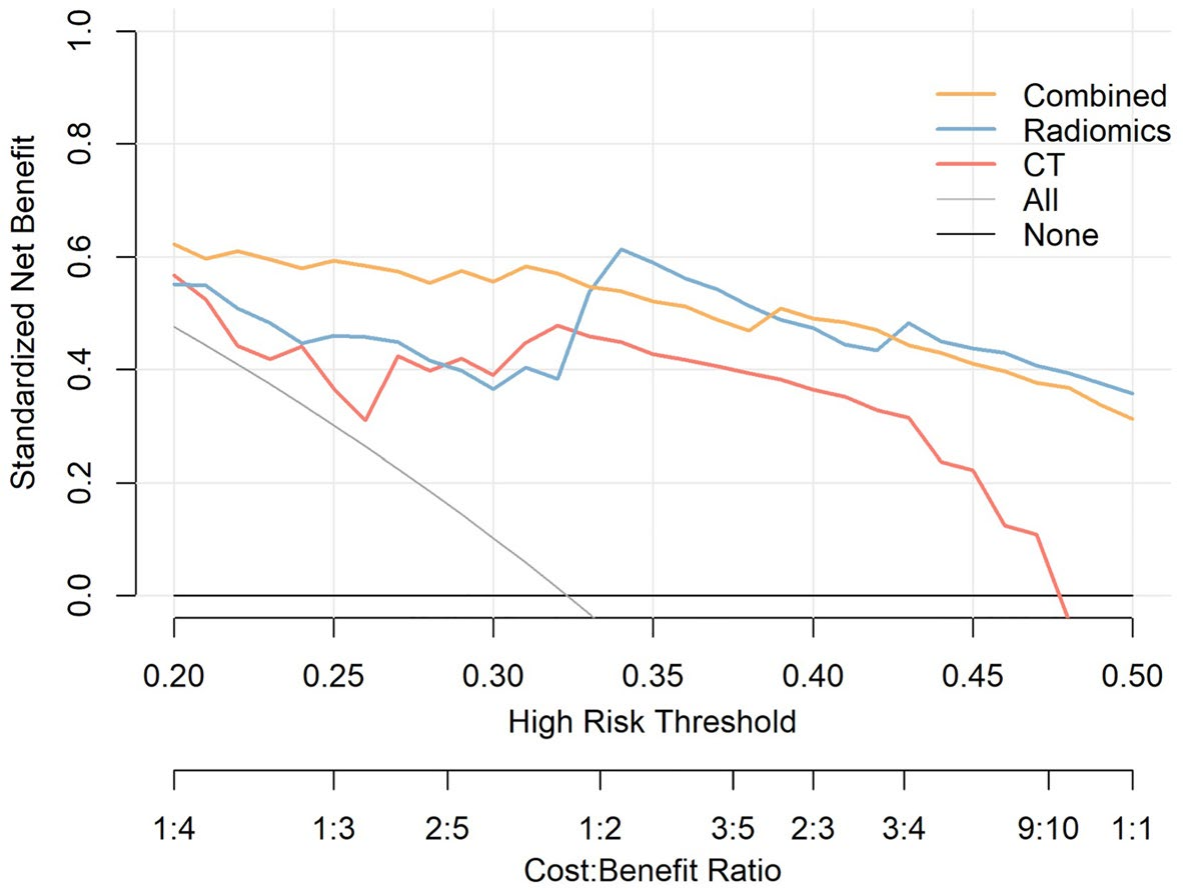
Decision curve analysis of the models. At the threshold of 0.2, the standardized net benefit achieved 0.6 in the test cohort. The DCA curve of the combined model is superior to that of the CT model across the whole range of reasonable risk thresholds, which was set to 0.20–0.50 in this study.

DCA of the combined model is shown in Fig. 6. All three models in this study have a higher NB than the default strategies. When comparing each model, the combined model presents the highest NB. It outperforms the CT model across the whole range of reasonable risk thresholds, which is set to 0.20–0.50 in this study. Additionally, the combined model was superior to the radiomics model at the 0.20–0.35 risk thresholds. The combined model and the CT model were comparable at the 0.35–050 risk thresholds.

**Figure 7:**
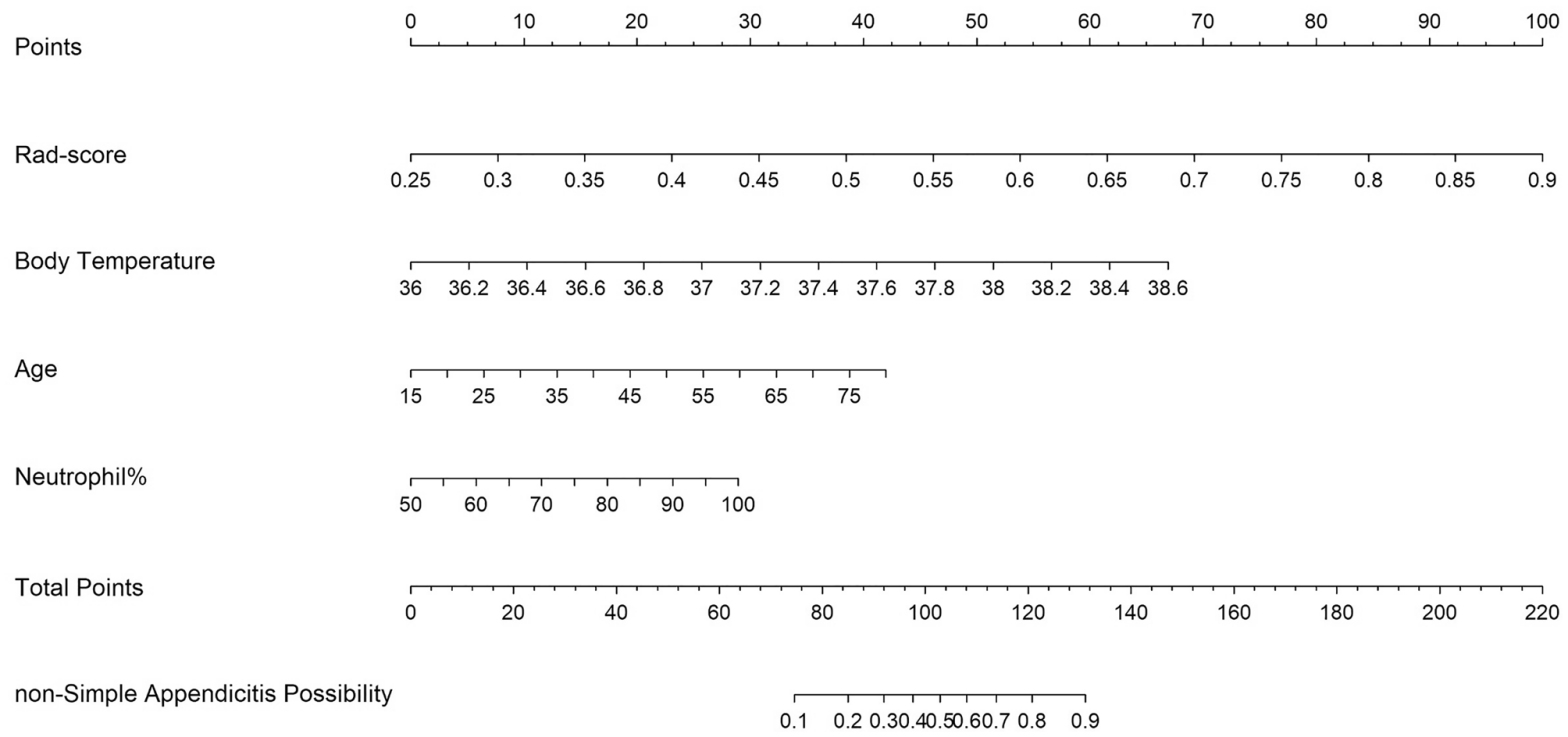
Nomogram of the combined model. A nomogram is a visual tool used to apply predictive models in clinical practice. It allows for the visualization of how different values of individual variables influence the final prediction outcome. In this nomogram, the top line (“Points” scale) serves as the reference for scoring points for each predictor from 0 to 100. The predictive variables, including Rad-score, body temperature, age, and neutrophil percentage, are displayed below with bars that represent their effect size, visually demonstrating the relative weight of each variable. These lines are plotted to assign points to each significant clinical characteristic. The sum of points from the above predictors can be checked on the “Total Points” scale, and then the corresponding predicted probability of non-simple appendicitis can be read from the bottom line (“non-Simple Appendicitis Possibility” scale). For example, using the following information from a patient to predict the likelihood of non-simple appendicitis: 35 years old, body temperature of 37.4 degrees Celsius, neutrophil percentage of 70%, and Rad-score of 0.60. Points are allocated accordingly on the “Points” scale: 35 years old corresponds to 12.922 points, 37.4 degrees Celsius corresponds to 36.024 points, neutrophil percentage of 70% corresponds to 11.575 points, and Rad-score of 0.60 corresponds to 53.846 points. Total Points = 12.922 (age) + 36.024 (body temperature) + 11.575 (neutrophil percentage) + 53.846 (Rad-score) = 114.367 points. We can locate 114.367 points on the “Total Points” scale by drawing a line down to the "non-Simple Appendicitis Probability” scale, indicating a 70% chance of a non-simple appendicitis case.

Finally, a nomogram of the combined model was plotted to represent our final model in this study (Fig. 7, Supplementary material).

## Discussion

Acute appendicitis is a common abdominal disease that can be treated with antibiotics or surgery^16^. Studies show that for simple cases, antibiotics are as effective as surgery^17^. Thus, we need an effective tool to differentiate types of acute appendicitis to avoid unnecessary surgical complications. Our study established three models (CT, radiomics, and combined) to differentiate non-simple appendicitis. DCA analysis showed that all models had a higher NB than default strategies, with the combined model having the highest NB. Therefore, our study demonstrated that it might be feasible to use a combination of clinical and radiomics information to identify non-simple appendicitis on CT images.

Acute appendicitis can be diagnosed using clinical indicators such as patient history, physical examination, and laboratory tests. Elevated leukocyte count is often associated with acute appendicitis, and the degree of elevation is positively correlated with the severity of the infection^16^. C-reactive protein (CRP) is also an objective indicator that can be used to predict infection and monitor treatment efficacy^17^. Hyperbilirubinemia can be observed in patients with complicated acute appendicitis, which may be caused by bacteria from the intestine to the liver parenchyma through the portal system^18^. Ulcerated appendiceal tissue in the septic or ruptured stage in complicated appendicitis releases more inflammatory factors and stimulates the liver to rapidly secrete large amounts of CRP^19^. In this study, univariate analysis showed that body temperature, CRP, WBC, NE, and bilirubin were significantly higher in patients with non-simple appendicitis. However, none of these indicators can be used as a definitive diagnostic tool.

CT scans are commonly used to diagnose acute appendicitis in clinical practice, but the interpretation relies on the radiologist's expertise. Radiologists can easily recognize typical findings associated with non-simple appendicitis, such as appendiceal intramural and extraluminal air, abscess, and confidently make a diagnosis of non-simple appendicitis. However, there are many findings that can present in both simple and non-simple appendicitis, such as thickness of the appendix, fecalith, thickening of the cecum wall, surrounding strand, pneumoperitoneum, and ileocecal lymph node enlargement. Previous studies also showed the relatively lower diagnostic efficacy of traditional CT features in this differentiation^20–23^. In this study, the multivariable regression results showed that the surrounding strand near the appendix and the thickening of the cecum were independent features associated with non-simple appendicitis, but these features can also be present in patients with simple appendicitis, making it difficult to determine the appropriate treatment using CT alone.

In clinical practice, doctors typically use a combination of clinical indicators and CT information to make a determination of simple versus non-simple appendicitis. However, this determination process is often highly subjective and not repeatable. This variability in diagnosis can lead to difficulty in determining the appropriate treatment and may result in a higher rate of complications or misdiagnosis. Therefore, many researchers are working to develop models that can integrate clinical indicators and CT information in a more objective and repeatable way to help improve the diagnosis of appendicitis and ultimately improve patient outcomes.

To extract CT information objectively and repeatedly, a radiomics method was used in this study. Radiomics is a technique that uses mathematical and statistical methods to analyze medical images and extract quantifiable data^19^. Many studies have reported that the radiomic model performs better than human radiologists in classification and prediction tasks. In this study, the training dataset was used to develop the models, and the test dataset was used to evaluate them. We first developed the radiomics model (using radiomic features from the CT images) and the traditional CT model (using CT visualization results by radiologists) in the training dataset, and the AUC of the radiomics model was higher than that of the CT model. Therefore, we selected the radiomic model to integrate with the clinical information for the development of the combined model. The predicted probability of the radiomics model, the Rad-score, was used as the input to the combined model. During the development of the combined model, the multivariable logistic regression showed that body temperature, age, neutrophil percentage, and the Rad-score were independently associated with non-simple appendicitis. Then, we evaluated the models in the test dataset. The DeLong test showed that the AUC of the combined model was significantly higher than that of the CT model, but the difference in AUC between the radiomics model and the CT model was not statistically significant due to the limited number of cases included in the study.

Recently, some researchers studied appendicitis on CT images using deep learning and radiomics methods^10–15^. Park et al.^12^ examined patients who underwent abdominal-pelvic CT scans due to acute abdomen and used 3D CNN to classify the appendix region. Their results demonstrated a diagnostic accuracy of 90% for acute appendicitis. Rajpurkar et al.^10^ proposed a deep learning model to detect abnormalities in the appendix region, achieving an AUC of 0.724–0.810. Noguchi et al.^11^ introduced a new method to evaluate the effectiveness of deep learning models in the context of the detection of acute appendicitis. The aim of the aforementioned studies was to differentiate between appendicitis and normal tissue. Lee et al.^13^ utilized a CNN to distinguish between appendicitis and diverticulitis, while Park et al.^15^ applied a CNN to differentiate between appendicitis, diverticulitis, and normal tissue. These studies all suggested the potential application of deep learning methods in evaluating acute appendicitis in different scenarios. However, none of them focused on distinguishing between simple and non-simple appendicitis, which differs from the scope of our study.

Liang et al.^14^ used a combined model of deep learning and radiomics to differentiate between complicated and uncomplicated acute appendicitis, achieving an AUC of 0.799. This study has a similar scope and yielded similar results to our research. The main distinction lies in the annotation of the ROI. In our study, we used a cube to label the appendix region in 3D CT images, while Liang et al.'s study needed radiologists to label the appendix region slice by slice on coronal CT images. We believe our annotation method is more convenient and feasible, making it potentially more applicable in clinical practice. Furthermore, slice-by-slice delineation of the ROI could be challenging in an emergency scenario. The radiomics features we extracted encompass not only the appendix region but also the surrounding area, whereas Liang et al.'s study only extracted features from the appendix. Since inflammatory changes in the appendix often extend to the surrounding area, incorporating features from the surrounding region of the appendix may be more reasonable. Similar studies have also demonstrated that ROIs that include peri-lesion or peri-organ areas can be used as ROIs for radiomics studies and produce accurate classification predictions^24^. However, the selection of the ROI has limitations. As the bounding box ROI was not restricted to the appendix, there is a possibility of overlap in findings related to other inflammatory conditions in the RLQ. To overcome this limitation, future studies should consider defining a more accurate ROI, possibly through the use of automatic segmentation methods. This would enable the isolation of the appendix from surrounding tissues and organs, facilitating the extraction of more specific features for precise appendicitis detection.

While the combined model proposed in this study demonstrates a certain level of capability in distinguishing non-simple appendicitis with relatively few false positives, it must be acknowledged that there are still a significant number of false negative cases. This is consistent with the results of other studies^14^. The presence of false-negative cases may lead to the incorrect administration of conservative treatment to patients who actually require surgery, potentially delaying the timing of the operation. Therefore, this should be given due consideration. When analyzing the false positive cases in this study, it can be observed that many cases leading to false positives exhibit insignificant inflammation changes on CT images. As a result, both CT visual assessment and radiomics may misclassify them as simple appendicitis. These patients primarily present with acute gangreanous appendicitis on pathology report, without signs of perforation or surrounding abscess. Recent studies have shown that this type of appendicitis responds well to conservative treatment, and the risk of misdiagnosis as simple appendicitis in such cases is relatively low^25^. On the other hand, studies have found that the use of contrast-enhanced CT scans can improve the identification of acute gangrenous appendicitis^26^. Therefore, in the future, we should further develop radiomic models based on enhanced CT scans in the hopes of reducing false negative results.

The quality of the data significantly impacts the accuracy of the radiomics models. High levels of data noise can decrease the model's robustness. The CT images used in this study have a median slice thickness of 1.25 mm and a relatively low signal-to-noise ratio, which could affect the results. However, these are the actual images used by radiologists in our clinical practice for diagnosis. Our model was trained using real-world data, and the results demonstrate that the radiomics model outperforms visual evaluation by radiologists, highlighting its value. In the future, we plan to collect data with slice thicknesses of both 1.25 mm and 5 mm from the same patients to train different radiomics models, compare the differences between them, and test the effect of slice thickness on the radiomics model.

In this study, a nomogram was created based on the combined model because it was found to be more accurate than either the CT model or the radiomics model alone. This nomogram incorporated the Rad-score, which is a predicted probability generated by the radiomics model, along with other clinical information, such as body temperature and age. Similar to other studies^24,27,28^, adding the Rad-score improves the accuracy of the classification task. The nomogram demonstrated that the Rad-score had a particularly strong effect on the diagnosis when it was in the range of 0.25–0.5, and when the Rad-score was above 0.7, it was highly indicative of non-simple appendicitis.

This study has several limitations. First, the study cohort was primarily composed of patients with non-simple appendicitis, as only those who underwent appendectomy were included. This resulted in an enrollment bias, as patients with simple appendicitis treated conservatively were not included, leading to a severe imbalance between positive and negative samples. In our clinical practice, the incidence of simple appendicitis is typically higher than or equal to that of non-simple appendicitis. The chances of a patient experiencing simple appendicitis and non-simple appendicitis were roughly equal in our clinical practice. This is the reason for considering a balanced 1:1 ratio of positive and negative cases in the training set. However, because simple appendicitis was matched to the training set, it resulted in a noticeable imbalance between positive and negative samples in the test set.To overcome this limitation, future studies should use a prospective cohort that includes both surgical and non-surgical patients to better reflect real-world clinical scenarios. Second, there is an inhomogeneous time interval between the CT scan and the operation. In this study, 95.5% of patients had a time interval of less than 24 h between CT scans and surgery. However, 4.5% of patients had a longer time interval between CT scans and surgery, and the condition of their appendix may have changed at the time of CT scanning, which may not reflect the actual disease status and could interfere with the study results. In the future, we should collect cases more strictly to further improve the model's performance. Third, only patients with unenhanced CT were enrolled due to the retrospective data. As so far, our results showed the relatively promising results of unenhanced CT with radiomics in the differentiation of appendicitis. However, we think that we also need to conduct more data with IV contrast for further study and may obtain better results. Another limitation is the manual annotation of the ROI for the appendix area. Although we have tested consistency among different annotators and ensured that the annotation results are reliable, the manual process may still be subject to error and consume a significant amount of human labor. In the future, an automated segmentation method should be developed to make the process more efficient and consistent. Finally, this study used data from a single center, and while it has demonstrated the feasibility of the radiomics method, it is not yet clear whether the model is robust and can be generalized to other settings. Further studies using multicenter data are needed to confirm the validity of the model.

## Conclusion

Through our study, we demonstrated the potential ability to integrate radiomic models with clinical information to identify simple appendicitis from non-simple appendicitis. The results of the study are preliminary, and further research is needed to validate the findings. After more studies, it is possible to determine the value of its clinical application.

### Supplementary Information

Supplementary Information.

## Data Availability

All these data and materials are available at any time from the corresponding author upon reasonable request.

## Abbreviations

PACS: Picture archiving and communication systems
CRP: C-reactive protein
WBC: White blood cell
NE%: Percentage of neutrophils
ROI: Region of interest
ICC: Intraclass correlation coefficient
LASSO: Least absolute shrinkage and selection operator model
ROC: Receiver operating characteristic
AUC: Area under the ROC curve
AIC: Akaike information criterion
PCCs: Pearson correlation coefficients
DCA: Decision curve analysis
CRP: C-reactive protein
