# Supplementary material for: Combination of clinical information and radiomics models for the differentiation of acute simple appendicitis and non simple appendicitis on CT images
Source: Sci Rep. 2024 Jan 22;14:1854. doi: 10.1038/s41598-024-52390-z (PMC10803326; doi:10.1038/s41598-024-52390-z)
Supplement: Supplementary file 1 — Supplementary Information. [file 41598_2024_52390_MOESM1_ESM.docx]

**Supplementary Materials**

**S1 Odds ratios in univariate and multivariate logistic regression analyses of the CT model**

The odds ratios in univariate and multivariate logistic regression analyses of the CT model are shown in Table S1.

**Table S1** Odds ratios in univariate and multivariate logistic regression analyses of the CT model

| **Variables** | **Univariable regression** | |  | **Multivariable regression** | |
| --- | --- | --- | --- | --- | --- |
|  | **OR (95% CI)** | ***P* value** |  | **OR (95% CI)** | ***P* value** |
| **Perforation** | 1.000 (0.116, 8.595) | 1.000 |  |  |  |
| **Abscess** | 1.000 (0.116, 8.595) | 1.000 |  |  |  |
| **Appendiceal Intraluminal Gas** | 1.728×10^7^ (0, NA) | 0.988 |  |  |  |
| **Pneumoperitoneum** | 5.984×10^6^ (0, NA) | 0.988 |  |  |  |
| **Appendix Wall Thickening** | 1.270 (0.581, 2.798) | 0.550 |  |  |  |
| **Peritonitis** | 1.646 (0.619, 4.584) | 0.323 |  |  |  |
| **Ileocecal Lymph Node** | 1.641 (0.740, 3.701) | 0.226 |  |  |  |
| **Fecal Retention** | 1.828 (0.810, 4.226) | 0.150 |  |  |  |
| **Thickness Of Appendix** | 3.434 (1.065, 12.879) | 0.050 |  |  |  |
| **Surrounding Strand** | 4.836 (1.605, 18.041) | 0.009 |  | 3.125 (0.956, 12.336) | 0.074 |
| **Cecum Wall Thickening** | 3.366 (1.512, 7.780) | 0.004 |  | 2.486 (1.052, 6.059) | 0.040 |

**S2 Parameters in the LASSO regression process**

The trend of the lambda and mean squared error during the fivefold cross validation of the LASSO regression is shown in Figure S1. The coefficients of the arguments with different L1 norm values during LASSO regression are shown in Figure S2. The coefficients of the arguments with different lambda values during LASSO regression are shown in Figure S3. The fraction deviance explained with different L1 norm values during LASSO regression is shown in Figure S4.


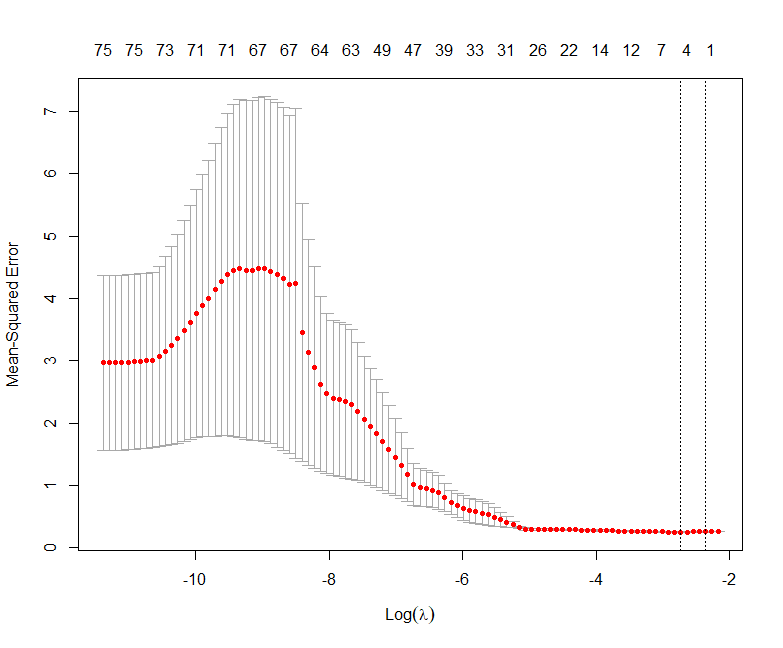
**Figure S1.** Trend of the lambda and m ean squared error during the fivefold cross validation of the LASSO regression


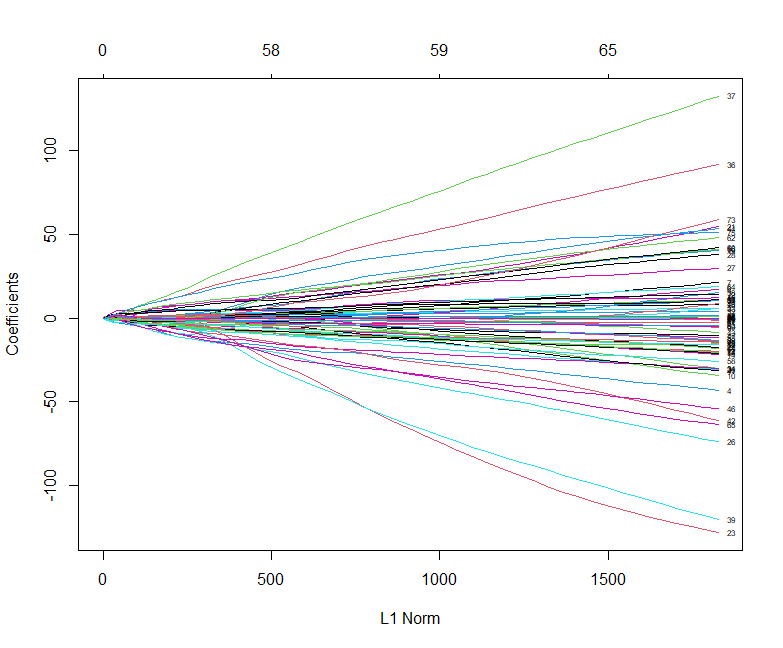
**Figure S2.** Coefficients of the arguments with different L1 norm values during LASSO regression


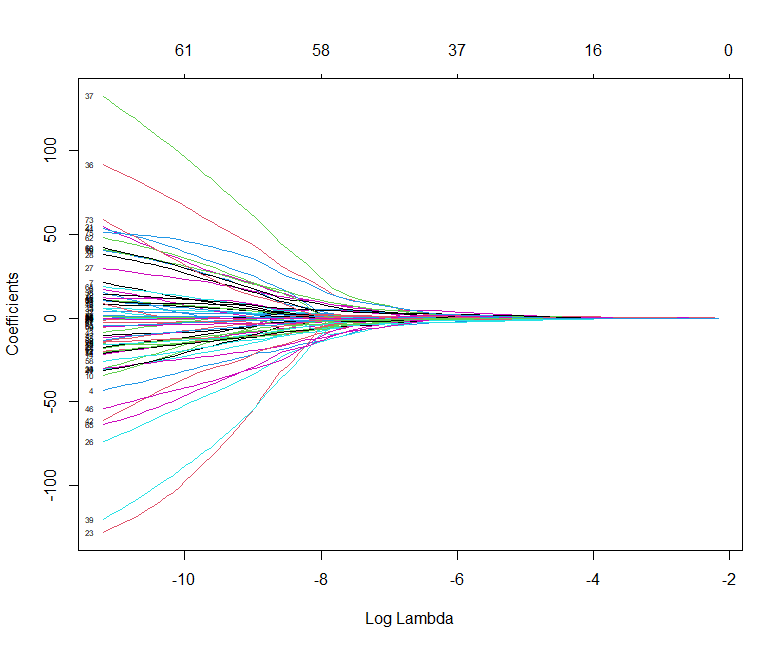
**Figure S3.** The coefficients of the arguments with different lambda values during LASSO regression


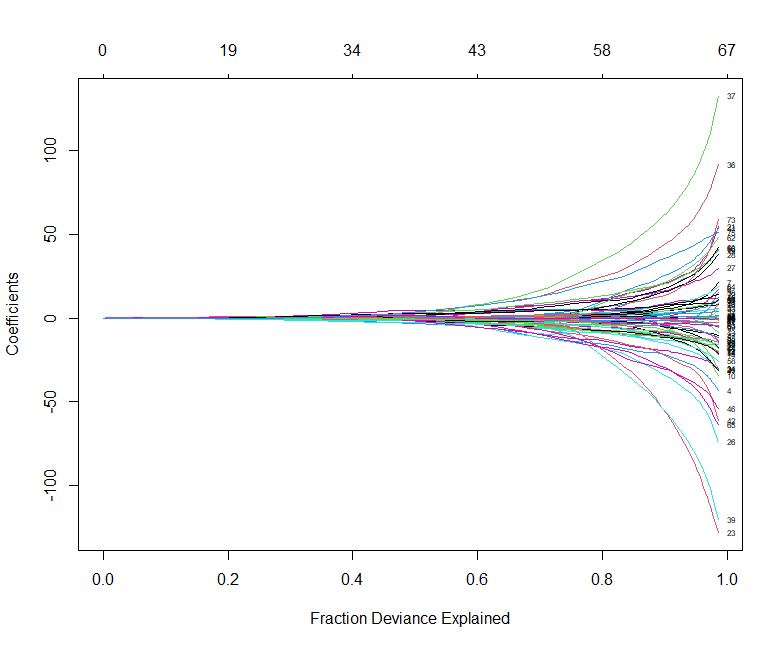
**Figure S4.** Fraction deviance explained with different L1 norm values during LASSO regression

...

**S3 Arguments in the LASSO regression model**

The arguments in the LASSO regression model are shown in Table S2.

**Table S2** Arguments in the LASSO regression model

|  | **Feature Type** | **Name** | **Value** |
| --- | --- | --- | --- |
| **Lambda** |  |  | 0.065 |
| **Intercept** |  |  | 0.007 |
| **Arguments** | GLDM | Dependence Variance | -0.204 |
|  | GLSZM | Large Area Emphasis | 0.243 |
|  | Shaped-based | Maximum 2D Diameter Column | 0.017 |
|  | Shaped-based | Minor Axis Length | 0.026 |
|  | Shaped-based | Surface Volume Ratio | <0.001 |

**S4 Precision-recall curve and the calibration curve**

After the best model was determined, the precision-recall curve and calibration curve were plotted for further evaluation of the best model. For the calibration plot, bootstrapping using 1,000 repetitions was used to obtain bias-corrected predictive accuracy measures of the models.

The PR curve of the combined model in the test cohort is shown in Figure S5, with an AUC of 0.974. The calibration curve is shown in Figure S6. Calculations using 1,000 bootstrap replicates showed that the mean absolute error of the calibration test was 0.015, the mean squared error was < 0.001, and the 90% quantile of the absolute error was 0.023.


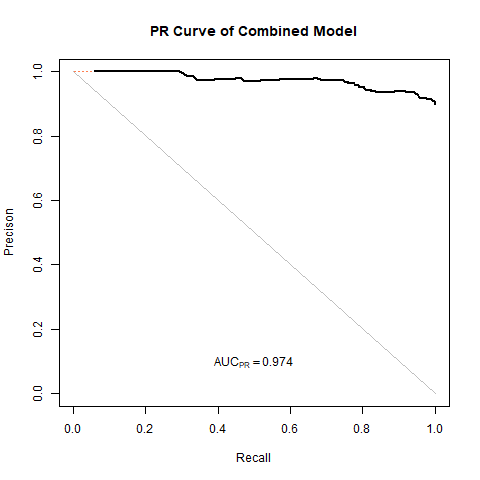
**Figure S5.** Precision-recall curve of the combined model in the test cohort, with an AUC of 0.974

**Figure S6.** Calibration curve of the combined model with 1,000 bootstrap replicates in the test cohort. Calculations using 1,000 bootstrap replicates showed that the mean absolute error of the calibration test was 0.015, the mean squared error was < 0.001, and the 90% quantile of the absolute error was 0.023.


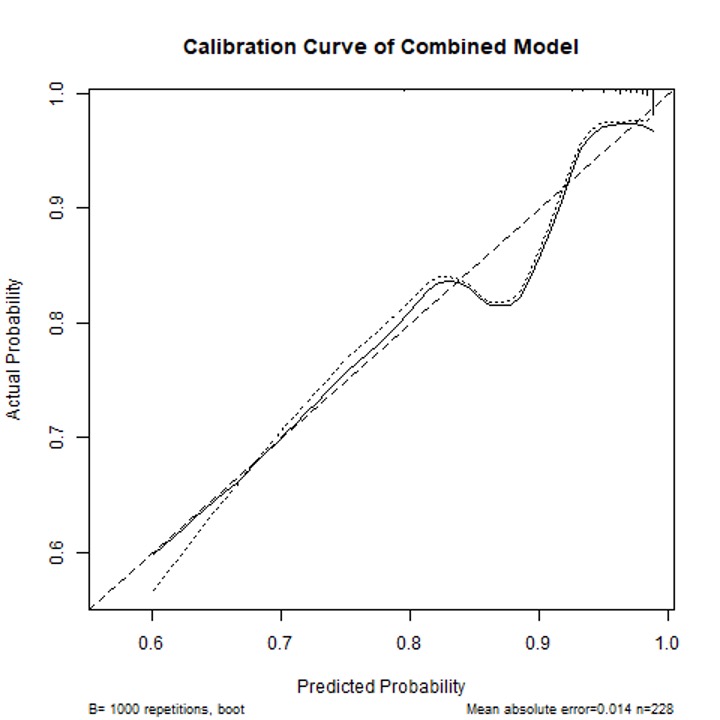


**S5 Points of the nomogram of the combined model**

The points of age, body temperature, neutrophil percentage, and Rad-score in the nomogram of the combined model are shown in Tables S3-S6. The total points vs the possibility of non-simple appendicitis are shown in Table S7.

**Table S3** Points of age in the nomogram of the combined model

| **Age** | **Xbeta** | **points** |
| --- | --- | --- |
| 15 | 0.753 | 0 |
| 20 | 1.0034 | 3.231 |
| 25 | 1.254 | 6.461 |
| 30 | 1.505 | 9.692 |
| 35 | 1.756 | 12.922 |
| 40 | 2.007 | 16.153 |
| 45 | 2.258 | 19.383 |
| 50 | 2.509 | 22.614 |
| 55 | 2.760 | 25.844 |
| 60 | 3.011 | 29.075 |
| 65 | 3.262 | 32.305 |
| 70 | 3.512 | 35.536 |
| 75 | 3.763 | 38.766 |
| 80 | 4.014 | 41.997 |

**Table S4** Points of body temperature in the nomogram of the combined model

| **Body Temperature** | **Xbeta** | **points** |
| --- | --- | --- |
| 36.0 | 71.940 | 0.000 |
| 36.2 | 72.340 | 5.146 |
| 36.4 | 72.740 | 10.293 |
| 36.6 | 73.139 | 15.439 |
| 36.8 | 73.539 | 20.585 |
| 37.0 | 73.939 | 25.732 |
| 37.2 | 74.338 | 30.878 |
| 37.4 | 74.738 | 36.024 |
| 37.6 | 75.138 | 41.171 |
| 37.8 | 75.537 | 46.317 |
| 38.0 | 75.937 | 51.463 |
| 38.2 | 76.337 | 56.610 |
| 38.4 | 76.736 | 61.756 |
| 38.6 | 77.136 | 66.902 |

**Table S5** Points of neutrophil percentage in the nomogram of the combined model

| **Neutrophil percentage** | **Xbeta** | **points** |
| --- | --- | --- |
| 50 | 2.247 | 0.000 |
| 55 | 2.472 | 2.894 |
| 60 | 2.697 | 5.788 |
| 65 | 2.922 | 8.681 |
| 70 | 3.146 | 11.575 |
| 75 | 3.371 | 14.469 |
| 80 | 3.596 | 17.363 |
| 85 | 3.820 | 20.256 |
| 90 | 4.045 | 23.150 |
| 95 | 4.270 | 26.044 |
| 100 | 4.495 | 28.938 |

**Table S6** Points of the Rad-score predicted by LASSO in the nomogram of the combined model

| **Rad-score** | **Xbeta** | **points** |
| --- | --- | --- |
| 0.25 | 2.987 | 0.000 |
| 0.30 | 3.584 | 7.692 |
| 0.35 | 4.182 | 15.385 |
| 0.40 | 4.779 | 23.077 |
| 0.45 | 5.377 | 30.769 |
| 0.50 | 5.974 | 38.462 |
| 0.55 | 6.571 | 46.154 |
| 0.60 | 7.169 | 53.846 |
| 0.65 | 7.766 | 61.538 |
| 0.70 | 8.363 | 69.231 |
| 0.75 | 8.961 | 76.923 |
| 0.80 | 9.558 | 84.615 |
| 0.85 | 10.156 | 92.308 |
| 0.90 | 10.753 | 100.000 |

**Table S7** Total points vs. possibility of non-simple appendicitis in the nomogram of the combined model

| **Total points** | **Possibility** |
| --- | --- |
| 74.582 | 0.1 |
| 85.024 | 0.2 |
| 91.964 | 0.3 |
| 97.654 | 0.4 |
| 102.875 | 0.5 |
| 108.096 | 0.6 |
| 113.785 | 0.7 |
| 120.725 | 0.8 |
| 131.167 | 0.9 |
